# Preservation of Vaccine by Cold below Freezing Point

**Published:** 1913-02

**Authors:** 


Preservation of Vaccine by Cold Below Freezing Point.—LeFavre, ibid., September 15, 1912, states that vaccine thus preserved was active after three years, but advises that it be used within two years.

